# Multidisciplinary approach to L3/L4 lumbar disc prolapse masquerading as focal limb myositis-a radiological challenge

**DOI:** 10.1259/bjrcr.20200126

**Published:** 2021-02-24

**Authors:** Ramandeep Singh Saini, Daniel Hurry, Rasvin Singh, Andrew Dapaah, Chetna Sharma, Denis Calthorpe, Virendra Jain, Ravi Kothari

**Affiliations:** 1 Royal Derby Hospital, Derby, UK

## Abstract

Prolapsed intervertebral discs are commonly associated with back ache and sciatica. Management is often conservative with analgesia and physiotherapy. Nerve root injections and discectomy procedures are used where conservative measures fail. Majority of patients present with symptoms of pain and motor weakness; however, a few can present as focal myositis of lower limb muscles in the distribution of radiculopathy. MRI scans of limbs are rarely done in these cases but if done can confound the radiologist. Our case report emphasize the importance of multidisciplinary approach for a L3 nerve radiculopathy with confounding clinical presentation of focal lower limb myositis of unknown etiology.

## Background

Prolapsed intervertebral discs are commonly associated with backache and sciatica. It is most commonly seen at L4–L5 and L5–S1 levels.^1^ Management is often conservative with analgesia and physiotherapy. Nerve root injections and discectomy procedures are used where conservative measures fail. The majority of patients present with symptoms of pain and motor weakness, however, a few can present as focal myositis of lower limb muscles in the distribution of radiculopathy. Various cases of S1 radiculopathy presenting as focal myositis have been described in the literature. We present a case of L3 radiculopathy with *a* confounding clinical presentation of lower limb myositis.

## Clinical details

A 69-year-old male presented to the emergency department with acute worsening of insidious onset backache and burning groin pain, radiating to the left inner thigh and shin. There were associated hypersensitivity and burning sensation along the medial aspect of the left thigh and leg. No associated numbness or weakness. No symptoms related to bladder or bowel. No history of trauma. No weight loss, change in appetite, or other systemic symptoms. Leg symptoms were severe compared to mild backache. He also had a past medical history of Rectal Cancer in 2009 which was treated by Chemotherapy (Capecitabine) and Radiotherapy followed by End Colostomy done in 2010 and treatment was completed thereafter. He also had hypertension and labyrinthitis, for which he takes regular Amlodipine and Statins. He smokes 15 cigarettes per day and drinks 20 units of alcohol per week. He was admitted under the general orthopedic ward which has visiting spinal consultants after review by core trainee Level 2 (4 years clinical experience) and Orthopedics registrar (with 6 years clinical experience).

On examination by registrar, he was found to have tenderness overlying the lumbar spine and left sacroiliac joint and was struggling to weight bear. He had some weakness with left hip flexion (Medical Research Council Grade 4) but power was otherwise normal throughout both lower limbs. There was hypersensitivity to light touch over the medial aspect of thigh and shin along with muscle wasting of the left thigh. His knee jerk reflex was also absent on the left.

We divided our differential diagnosis as (*a) Spinal* - lumbar nerve root impingement, *(b) Peripheral* - compressive pelvic pathology (*c) Post-radiotherapy/Chemotherapy*, (*d) Nerve infiltration* – due to proximity of previous rectal cancer, (*e) Neuromuscular junction/Muscular* - primary muscle pathology, *e.g*. myositis, (*f) Paraneoplastic* – given past medical history of rectal cancer.

**Figure 1.**
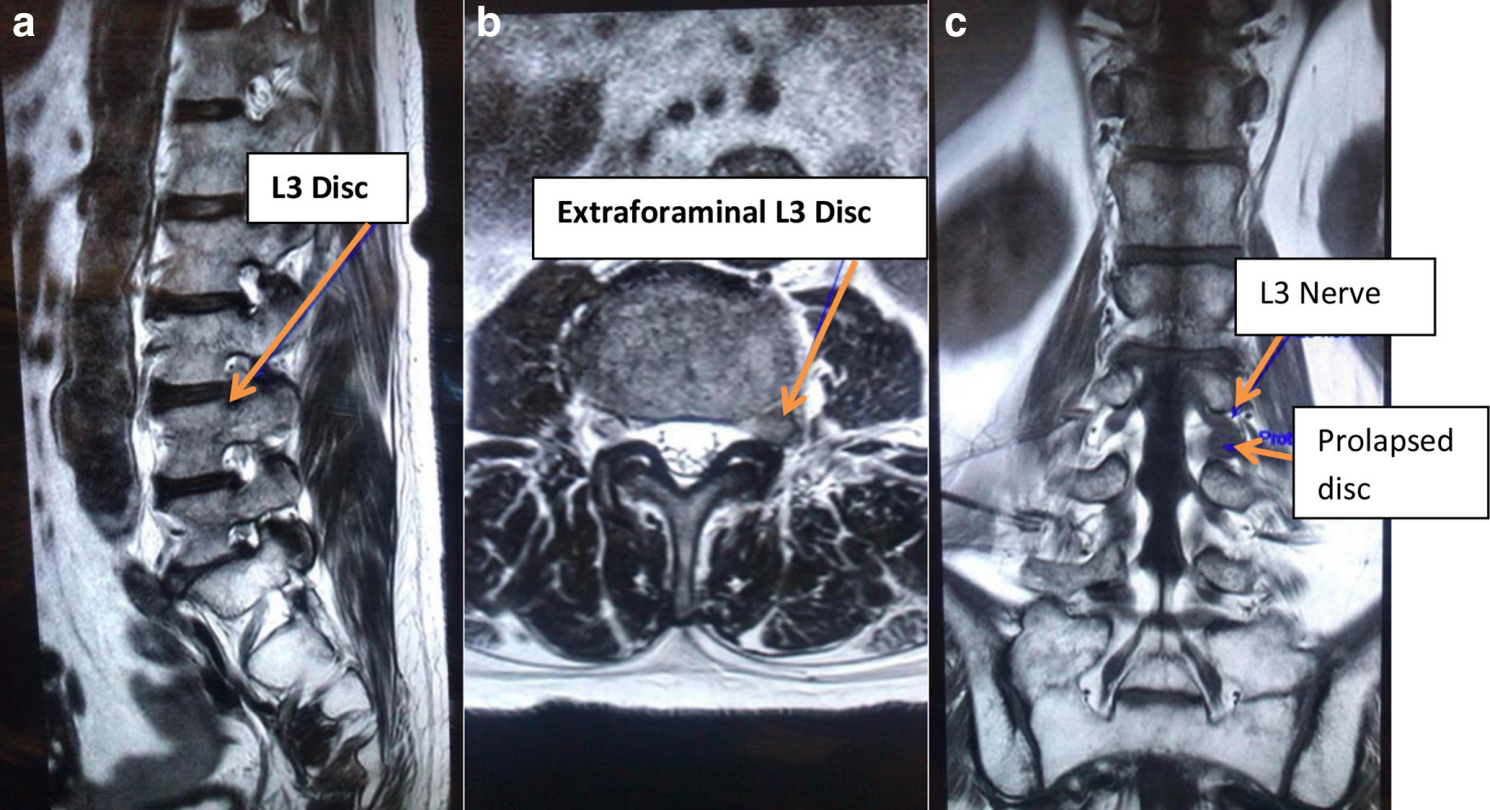
MRI scan LS spine. (a–c) showing Sagittal T2, Axial T2, and Coronal T1 sections of LS MRI respectively. Extraforminal disc can be seen on 1b and 1c. At L3-L4 disc level lifting of the L3 Nerve root due to disc in the recess between cauda equine and L3 nerve root (also called axillary presentation of disc) can be seen in (c). (a) Sagittal T2 section MRI. (b) Axial T2 section. (c) Coronal T1 section. LS, Lumbosacral.

**Figure 2.**
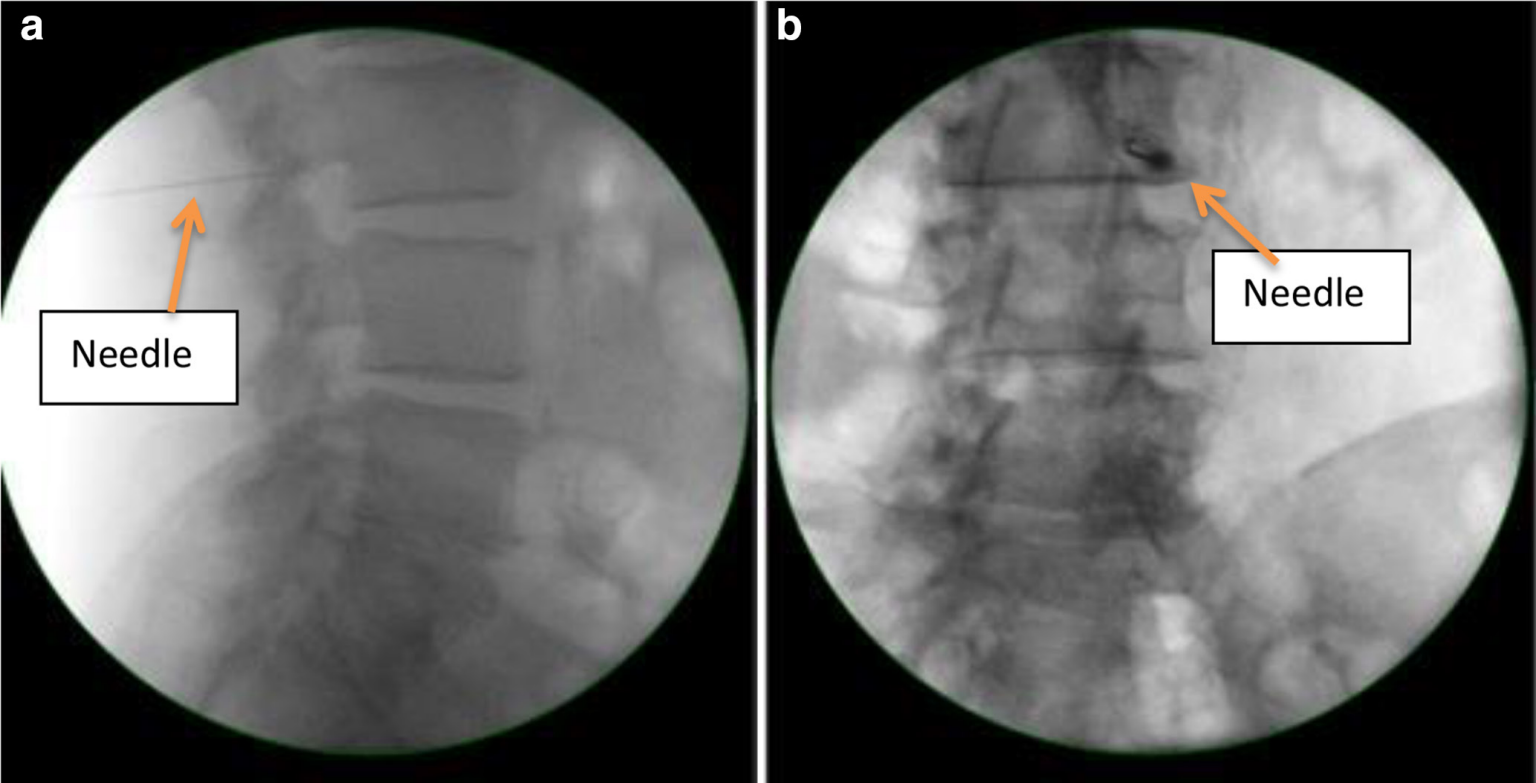
Diagnostic L3 Nerve root block using CARM image to confirm level. (a) Sagittal image using C-arm. (b) Coronal image using C-arm. L3 vertebral body is localized in Sagittal and Coronal images using C-arm machine. Needle can be visualized in sagittal and coronal section under L3 vertebral body pedicle. Nerve root block is given thereafter.

MRI of the lumbosacral spine revealed a left-sided extraforaminal disc prolapse at L3/L4 with suspected impingement of the left exiting L3 nerve without compression of descending L4 nerve root (Figure 1a–c). He was reviewed and examination findings of registrar were confirmed by a Spinal consultant with more than 30 years of clinical experience and care was transferred to a tertiary center with spinal facilities. Diagnostic L3 nerve root block offered only modest temporary respite (Figure 2). The case was discussed in Spinal MDT (multidisciplinary team meeting) among Radiology, Oncology, and Spinal consultants. As symptoms failed to settle on expectant management, further investigations and imaging with Nerve Conduction velocity (NCV)/Electromyography (EMG) and MRI of the lower limb and pelvis were carried out keeping differentials in mind. An MRI scan of the pelvis and ultrasound of the groin done by a Consultant Radiologist with more than 10 years of experience excluded local pathology.

**Figure 3.**
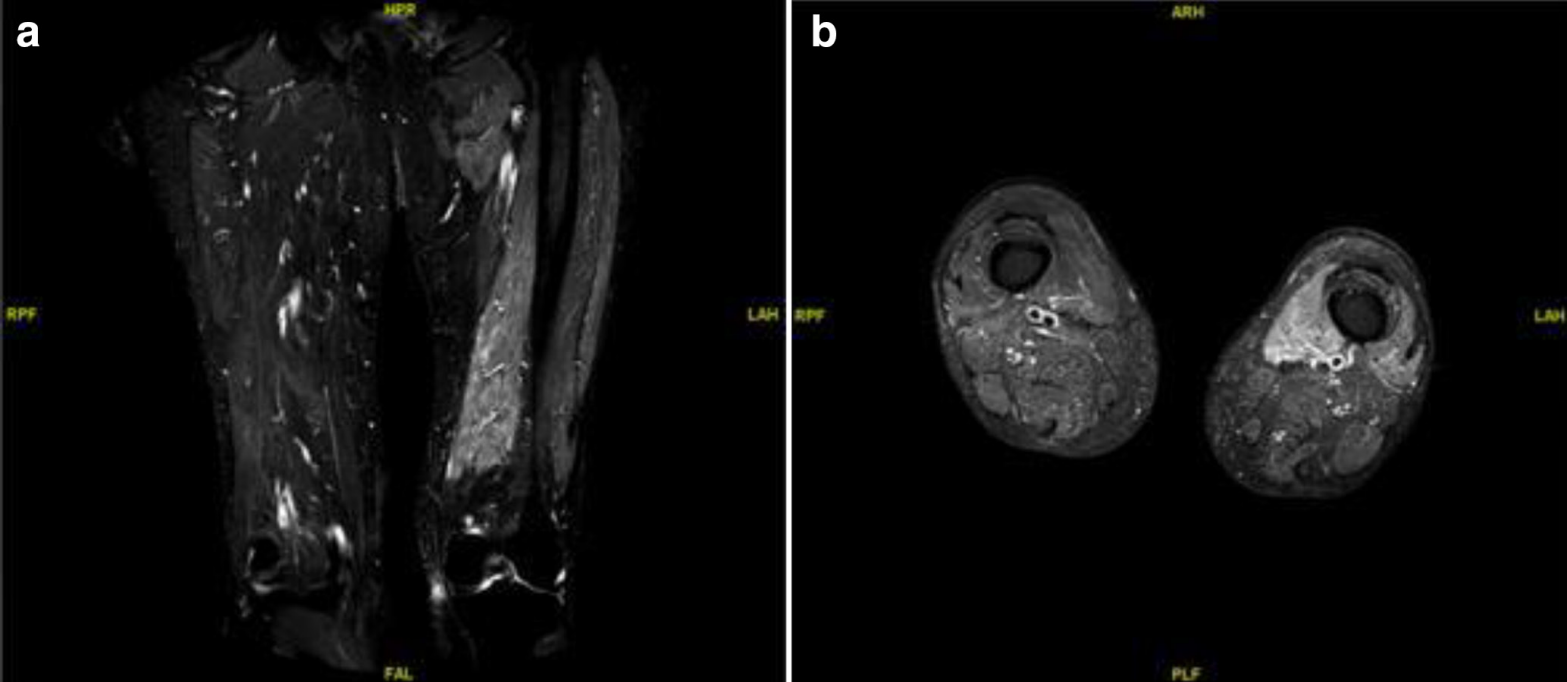
MRI of both thighs STIR sequence. (a) Coronal section STIR MRI both thighs. (b) Axial section STIR MRI both thighs. MRI of both thigh and leg reported by MSK Radiologist as marked oedema of Vastus Medialis, Adductor compartment, and even going to Tibialis Anterior of left side. Myositis of unknown aetiology suggested. Potentially due to inflammatory or ischemia process. Infectious aetiology is possible but less likely. STIR, short tau inversion recovery

MRI scan of thigh and legs (Figure 3) reported by an experienced Musculoskeletal Radiology consultant confirmed discrete myositis of unknown etiology. Potentially inflammatory or ischemic. Infectious etiology possible but less likely involving Vastus Medialis, Adductor compartment, and Tibialis Anterior muscles. No associated collection or surrounding soft tissue inflammatory changes noted.

Low dose steroid was started for myositis, as there was no evidence of infection but the response was minimal. NCV and EMG suggested denervation changes in Vastus Medialis, Adductor compartment, and Tibialis Anterior. EMG revealed dysfunction of upper lumbosacral plexus (Femoral and Obturator nerves). It also revealed bilateral lower limb sensorimotor axonal neuropathy (Common Peroneal and Tibial), which was suspected of chemotherapy-induced. The conclusion of the report was? Inflammatory v/s infiltrative pathology of L3 and L4 ± L2 with the suggestion that MRI can show diffuse muscle edema in the acute/sub acute phase of denervation.

A Rheumatology Consultant opinion was taken due to the presence of myositis on MRI and modest response in symptoms to nerve root block and steroids. However*,* in absence of joint swelling and twitches/cramps, normal serum CK levels (58–73), and absence of myositis changes on nerve conduction velocity studies the experienced consultant was not fully convinced with the diagnosis of myositis and suggested further for a neurologist’s opinion.

The patient was extensively investigated by Consultant Neurologists to rule out autoimmune, vasculitic, and atypical infection. CT-Chest–Abdomen-Pelvis and MRI Pelvis/Lumbar plexus were also done without adding much to this challenging case.

This case was again discussed in MDT and it was found that exhaustive investigations failed to rationalize the case in terms of systemic pathology or the disc prolapse. This may have been a presentation of muscle change secondary to denervation. Management was commenced on empirical analgesia, low dose finite steroid regime, spinal and neurology follow up with interval MRI scanning.

**Figure 4.**
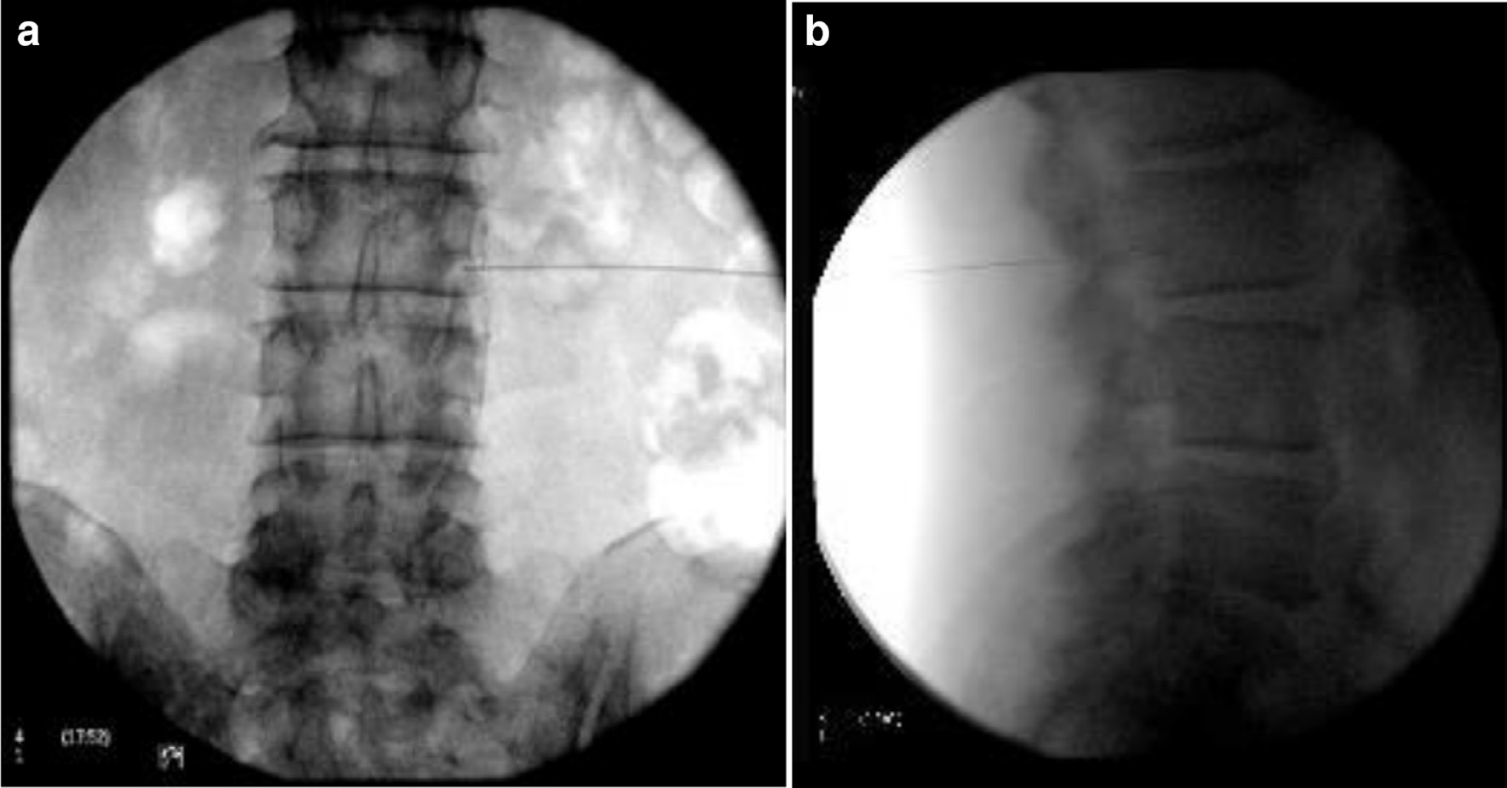
Repeat L3 Nerve root block using C-arm X-ray image in theatres. (a) Coronal image C-arm. (b) Sagittal image C-arm. Figure 4 shows repeat L3 Nerve root block images from C-Arm. Level was localized in Coronal (a) and Sagittal images (b) before giving injection.

Further, repeat L3 nerve root block ± discectomy recommended. Repeat L3 Nerve root block (Figure 4) was performed with nearly 60–80% improvement in symptoms.

**Figure 5.**
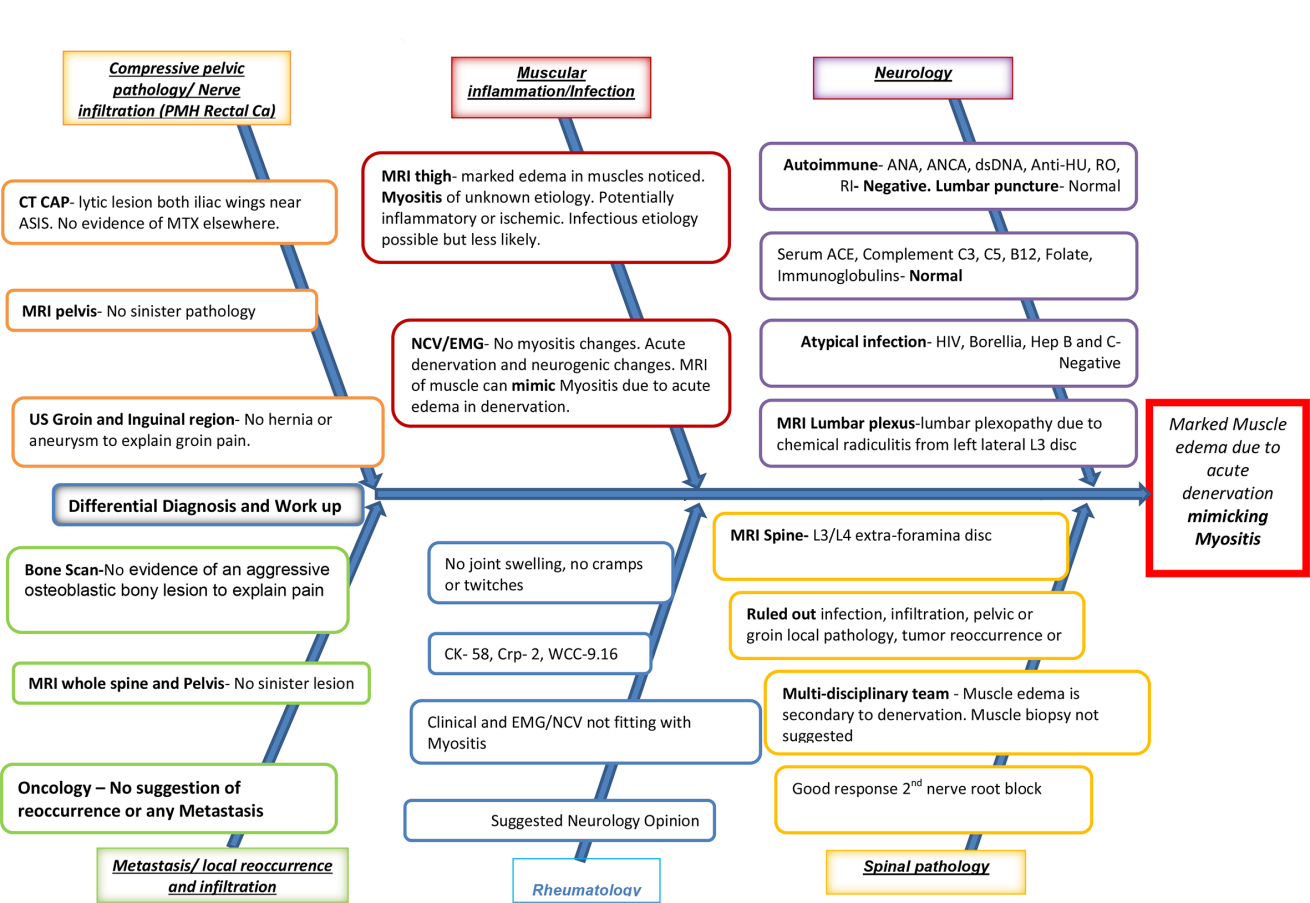
Fish Bone Diagram showing extensive work-up and multidisciplinary approach to rule out potential differential diagnosis.

The last OPD follow-up showed no further deterioration in symptoms. The latest interval MRI scan showed left-sided lumbar plexopathy change (L3 mainly, some in L4 and Obturator nerve) and denervation change, grossly unchanged in comparison with the previous study. Appearances are likely to be due to chemical radiculitis from a small left lateral L3 disc protrusion. Extraforaminal discectomy was offered to the patient if symptoms reoccur or worsens, though the patient asked for some more time to make an informed decision. A fishbone diagram below shows the exhaustive work-up done for this patient. (Figure 5)

## Discussion

Radiculopathies are commonly seen in the L5/S1 level and are usually treated conservatively. L3/L4 extraforaminal disc presenting as L3 radiculopathy is itself a rare entity.^1^ Analgesia and physiotherapy are the first lines of therapies offered to patients. Selective nerve root blocks and discectomy procedures are usually reserved for refractory cases. We followed the same principles in our case and offered the patient analgesia first followed by the L3 nerve root block as per NICE guidelines.^2^ The absence of response to analgesia and selective L3 Nerve root block leads to a battery of investigations and several discussions at MDT meetings for our case.

Myositis is commonly associated with myopathic disorders, however, it can be rarely seen with neurogenic disorders. In polyneuropathy or anterior horn cell disease, the presentation is usually bilateral. Unilateral myositis secondary to a neurogenic cause is rare and most commonly has been described with S1 radiculopathy. It has also been reported with L5 radiculopathy, polyneuropathy, and poliomyelitis.^3^ Since the detection of the first case of focal myositis in a patient of S1 radiculopathy by Krendel in 1992, total nine cases of such focal myositis related to S1 radiculopathy have been described in the literature until 2013 by Hemmi et al, however, none of the cases of L3–4 radiculopathy has been described in literature till date.^4,5^

No evidence of infection was found in our case with a normal range of inflammatory markers. Chemoradiation causing myopathy of hip muscles was reported by Florczynski et al^6^ in a case of adenocarcinoma of the rectum. That happened 5 month post-radiotherapy and 2 month post-chemotherapy completion in their case. We ruled out chemoradiation as the possible cause of myositis due to the onset of symptoms after 9 years of treatment completion in our case.

Steroid treatment is supported by literature without any other intervention.^7^ A few of the cases described happened after laminectomy at L5–S1, responded well to steroids.^8^

The definite cause of myositis in radiculopathy is poorly understood. Gross et al^8^ did a systematic review and case report search and found six cases of S1 radiculopathy associated with myositis in 2008. Although the mechanism behind muscle inflammation remains largely unclear, they strongly supported the notion that denervated muscles can develop inflammatory responses.^8^ In an animal experimental study done on the denervated muscle of mice, Kampmann et al^9^ demonstrated mononuclear inflammatory response with CD8 lymphocytes. Another theory is the mechanical process in terms of repeated microtrauma due to walking on denervated muscle, which could be the cause of myositis.^5^

Although EMG is the gold-standard MRI which is a very useful investigation in muscle pathologies like myositis. MRI is a non-invasive tool that can identify muscle groups even not accessible to EMG. MRI detects edema with T2 and STIR uptake in acute denervation (<1 month) in muscle groups like in our case. The radiologist could be the first person to identify the muscle groups and can classify the stage of muscle denervation as acute (edema with T2 and STIR uptake), subacute (further increase in extracellular water), and chronic (atrophy, fat infiltration). Having a panoramic view of muscles involved in a limb nerve involvement can be identified.^10^

Smitaman et al^11^ classified muscle pathologies into four patterns on MRI scans. (*a*) Abnormal anatomy with normal signal intensity (primary disorders/anomalies or post-operative), (*b*) edema/inflammation related to increases in water content (Inflammatory, infection, drug/radiation-induced, infection), (*c*) intramuscular mass causing anatomic distortion (metastasis, neoplasm, hematoma, etc.) or (*d*) atrophy resulting in tissue loss, usually accompanied by fatty replacement (Congenital, end-stage inflammation, denervation). Often, the presentation is mixed, *e.g.* acute denervation starts with edema and later progresses to the atrophy stage. Hence, edema was recognized in our case where a bilateral lower limb MRI scan was done in the acute denervation phase. Edema in itself is very non-specific, therefore diagnosis needs a multidisciplinary approach as in our case.

Empirical analgesia and steroids did not settle the leg symptoms in our case. In an MDT meeting, it was decided not to perform invasive muscle biopsy for our patient. Hence, denervation-related muscle edema should be a correct term rather than myositis for our case. Focal muscle changes are infrequently identified in association with lumbar disc prolapse, as we rarely image the lower limbs. However, if a lower limb scan shows features of possible focal myositis or edema, a spinal cause should be kept in mind as happened in this case. Ours is the only case, where we targeted spinal cause in first place for this L3/L4 disc pathology masquerading as myositis/denervation muscle edema. Unfortunately, there was no response to the first nerve root block given at the L3 level. Therefore, failure to respond doesn't mean wrong diagnosis/treatment. The second nerve root block did provide good relief of acute symptoms and allowed the patient to weight bear again. Although the explanations for this could be multifactorial, *e.g.* the natural history of disc radiculopathies cannot be ignored where symptoms can remit in 6–8 weeks. The second nerve root block was given around this period, and the patient was under cover of empirical analgesia and finite low dose steroid regimen.

## Conclusion

L3 radiculopathy has never been described in the literature as a cause of denervation related muscle edema. The pathophysiology is multifactorial, which includes denervation inflammatory response in muscles and repeated microtrauma to denervated atrophic/weakened muscles. If MRI of the limb is performed, it can show features of myositis which can confound the radiologist if the spinal cause is not kept in mind.

## Learning points

1. L3 radicular pain is uncommon but can be a cause of focal lower limb pain.
2. MRI of the limb can show diffuse edema in acute/subacute denervation, therefore spinal cause should be considered.
3. MDTs (spinal, rheumatology, neurology, radiology, and oncology) are required for the care of the patient.
4. Failure to respond to treatment doesn’t necessarily mean the wrong diagnosis, but even response to treatment should not deter further work-up.
